# Phosphatidylinositol-4-phosphate joins the dance of plant autophagosome formation

**DOI:** 10.1080/15548627.2022.2132042

**Published:** 2022-10-10

**Authors:** Rodrigo Enrique Gomez, Julie Castets, Josselin Lupette, Clément Chambaud, Jérôme Joubès, Amélie Bernard

**Affiliations:** Laboratoire de Biogenèse Membranaire, UMR 5200, CNRS, Univ, Bordeaux, Aquitaine, France

**Keywords:** Arabidopsis, autophagosomes, environmental stresses, lipids, phosphatidylinositol-4-phosphate, plasma membrane

## Abstract

In plants, macroautophagy/autophagy is a key mechanism that contributes to their ability to cope with a wide range of environmental constraints such as drought, nutrient starvation or pathogen resistance. Nevertheless, the molecular mechanisms of plant autophagy, and notably that of autophagosome formation, remain poorly understood. As the starting point of our recent paper, we considered the potential functional contribution of lipids in the numerous membrane-remodeling steps involved in this process. By combining biochemistry, genetics, cell biology and high-resolution 3D imaging, we unraveled the function of the lipid phosphatidylinositol-4-phosphate (PtdIns4P) in autophagy in *Arabidopsis thaliana,* thus providing novel insights into the assembly of autophagosomes in plant cells.

Lipids are critical components of biological membranes, both as building blocks and as key functional actors defining their structure, identity and activities. Although autophagosome formation relies intensely on membrane modeling and re-modeling events, from the nucleation of the phagophore to its expansion and closure, we critically lack knowledge concerning the nature and function of lipids in this process in plants.

Prior to this study [1], we performed a lipid-inhibitor screen in order to identify lipids that are critical for autophagy in the model plant *Arabidopsis thaliana*. Autophagy was induced by nutrient starvation and the impact of several lipid inhibitors was assessed on autophagy flux in root cells. These experiments notably showed that pharmacologically blocking the activity of PtdIns 4-kinases (PtdIns4K), thus depleting the cells of PtdIns4P, results in a large block in autophagy degradation. To investigate the role of PtdIns4P in plant autophagy, we monitored the subcellular localization of several ATG8 isoforms to visualize autophagic structures in regard to PtdIns4K activity. We found that in the absence of PtdIns4P synthesis, the number of ATG8 puncta drops drastically, indicating a defect in the formation of autophagic structures. This reduced number of autophagosomes is observed regardless of the autophagy-inducting conditions tested (nutrient stress, TOR inhibition) or the ATG8 isoform observed (ATG8a, ATG8e, ATG8f), highlighting the general importance of PtdIns4P in autophagosome formation. Additionally, our biochemical analyses showed that PtdIns4K activity is required for the proper lipidation of ATG8s and their recruitment to the phagophore membrane. In contrast, we showed that the membrane association of selected ATG proteins (ATG4, ATG5 and ATG18), working upstream of ATG8 in autophagosome formation, is not compromised upon PtdIns4K inhibition. From these results we concluded that PtsIns4K is not required for the nucleation of an early phagophore but is essential for its structure/assembly and expansion, notably by promoting ATG8 lipidation.

PtdIns4P is a negatively charged phosphoinositide that plays essential roles in multiple cellular processes. Notably, PtdIns4P shapes the identity of the membranes it is part of, by recruiting specific sets of polybasic proteins through electrostatic interactions. In that context, we initially hypothesized that PtdIns4P is present at the phagophore membrane and could participate in autophagy by mediating the association/dissociation of key molecular players of autophagosome formation. To explore the presence of PtdIns4P on autophagic structures, we performed fluorescence microscopy analyses using specific lipid biosensors, which show an accumulation of a PtdIns4P probe on autophagic bodies (the single-membrane vesicles present in the vacuole lumen resulting from the fusion of an autophagosome with a vacuole) but do not show an enrichment of the PtdIns4P probe on early autophagy structures. Yet, in these confocal analyses, we often find autophagic structures, with a larger proportion of phagophores than autophagosomes, close to the plasma membrane (PM).

In order to gain more definitive insights about the subcellular environment of autophagosome formation, we performed correlative light-electron microscopy (CLEM) using plants expressing GFP-ATG8a and a PtdIns4P-specific probe. As expected, we found that the vast majority of autophagic structures are in very close vicinity to the endoplasmic reticulum (ER) which supports the idea that most autophagosomes emerge from this compartment. Additionally, we found that a significant amount of autophagic structures (about 40%) are in proximity to the PM or the Golgi/trans-Golgi network (TGN), the two main reservoirs of PtdIns4P in plant cells; although PtdIns4P displays a much higher concentration at the PM compared to the Golgi-TGN. To date, three *bona fide* PtdIns4P-producing PtdIns4K enzymes have been characterized in Arabidopsis, which are thought to promote the establishment of such pools: PI4Kα1 at the PM and PIKβ1 and PI4Kβ2 at the TGN. While knocking out PI4Kβs causes no effect on autophagy in our experimental conditions, we showed that knocking down PI4Kα1 alters autophagy degradation and plant recovery from prolonged starvation, revealing the contribution of PI4Kα1 in autophagy.

Considering the implication of PI4Kα1, the proximity of autophagy compartments to the PtdIns4P-enriched PM and the requirement of PtdIns4P for autophagy, we thus proposed a model (Figure 1) whereby the PM acts as a platform for autophagosome formation by providing PtdIns4P during the early stages of phagophore expansion. Although the molecular mechanism by which PtdIns4P controls the proper assembly and expansion of the phagophore remains to be characterized, we speculate that PtdIns4P from the PM recruits an essential component(s) of the autophagy machinery upstream of ATG8 lipidation. Notably, PtdIns4P could participate in the establishment of contacts between the PM and the ER that are suggested to be required for autophagosome formation in plants. Future research will aim at identifying this PtdIns4P-effector protein(s) to better understand the mechanism by which PtdIns4P controls plant autophagy. In addition to the function of PtdIns4P in the early stages of autophagosome formation that we uncovered in our study, whether PtdIns4P also participate at later stages of the pathway remains unexplored. PtdIns4P can be rapidly interconverted to other phosphoinositide species, thus providing a fast and acute input in the dynamics of membrane compartments. Therefore, a changing composition of PtdIns4P at autophagic structures may be required to support distinct steps of the autophagy process. Our results show no noticeable accumulation of PtdIns4P in early autophagic structures, yet we found that PtdIns4P accumulates on autophagic bodies, which might be reminiscent of the function of PtdIns4P in autophagosome-lysosome fusion previously described in other organisms. Addressing the role of PtdIns4P in autophagosome maturation and autophagosome-vacuole fusion should be the subject of future research.

**Figure 1.**
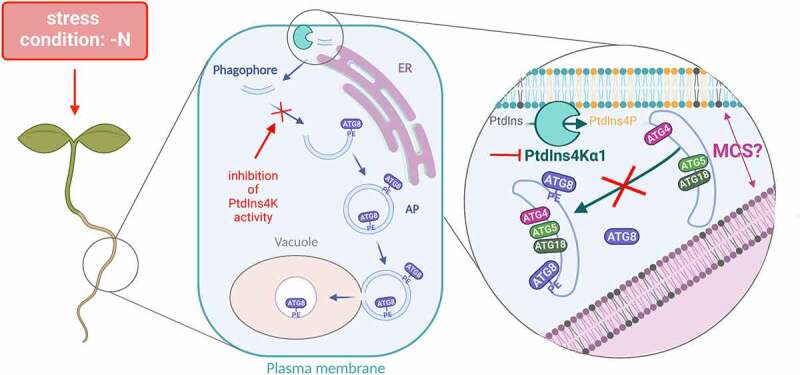
The anionic lipid PtdIns4p is crucial for the expansion of early autophagic structures into autophagosomes. Schematic view of the proposed role of PtdIns4p in autophagy in the root cells of *Arabidopsis thaliana* upon nutrient starvation (−N). Autophagosome (AP) biogenesis begins with the nucleation of the phagophore in proximity to both the endoplasmic reticulum (ER) and the plasma membrane, at putative membrane contact sites (MCS). The plasma membrane production of PtdIns4p by the phosphatidylinositiol 4-kinase PI4Kα1 is required for autophagy. Inhibiting PtdIns4k activity hampers the lipididation of ATG8 to phosphatidylethanolamine (PE) and its recruitment to the early autophagy compartment thereby disrupting the expansion of the phagophore and autophagosome formation.

In sum, our study unraveled new insights about the membrane dynamics and mechanisms involved in autophagosome formation, a critical process for plant acclimation to stress. As such, we hope that our results can provide keys to understand and to deal with the effects of environmental changes on plant physiology and crop production.
